# What Makes Household Sanitation Systems Resilient to Floods? Evidence from Ethiopia, Uganda, and Nepal

**DOI:** 10.1021/acsestwater.5c01055

**Published:** 2026-02-17

**Authors:** Jeremy Kohlitz, Abraham Geremew, Kenan Okurut, Prativa Poudel, Anish Ghimire, Anisha Nijhawan, Alejandro Valenzuela, Jay Falletta, Anjali Manandhar-Sherpa, Juliet Willetts, Guy Howard

**Affiliations:** † Institute of Sustainable Futures, 1994 University of Technology Sydney, 235 Jones Street, Sydney, New South Wales 2007, Australia; ‡ Department of Environmental Health, College of Health and Medical Sciences, Haramaya University, P.O. Box 138, Dire 138, Dawa, Ethiopia; § 108107 Kyambogo University, P.O. Box 1, Kyambogo, Kampala, Uganda; ∥ Department of Environmental Science and Engineering, 92961 Kathmandu University, Dhulikhel, 45200, Nepal; ⊥ Asian Institute of Technology, 8 Moo 9, Km. 42, Paholyothin Highway, Klong Luan 12120, Pathum Thani, Thailand; # School of Civil, Aerospace and Design Engineering and Cabot Institute for the Environment, 1980 University of Bristol, Bristol BS8 1TR, U.K.

**Keywords:** toilets: latrines, flooding, climate change, vulnerability, Africa, Asia, LMICs

## Abstract

Climate change is influencing precipitation events and patterns, leading to more frequent and severe flooding in many regions worldwide. In low- and middle-income countries, concerns about worsening floods disrupting access to safe sanitation for households are driving discussions about how to make sanitation systems more resilient. Much of this discourse relies on context-specific experiences or theory. This study surveyed 1,429 households in Nepal, Ethiopia, and Uganda to identify attributes linked to poor sanitation outcomes due to flooding to better inform sanitation planning and policy. Logistic regression was used to examine correlations between household and sanitation-system characteristics and (1) sanitation system failures and (2) adoption of open defecation following floods. The findings suggest that exposure to flooding significantly increases household sanitation system failures and that the quality of the construction and maintenance of sanitation facilities is correlated with these failures. Living in rural areas, using poor-quality latrine slabs, and discomfort in using a neighbor’s toilet were correlated with open defecation following flood damage to household sanitation systems. These findings support a policy focus on well-built, well-maintained sanitation in flood-prone areas and the provision of alternatives when facilities fail. Other commonly recommended measures, such as raising latrines, were not found to correlate with outcomes and require further investigation.

## Graphical abstract

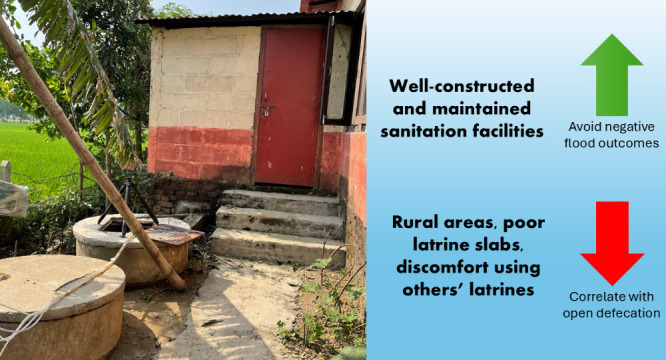

## 1 Introduction

Global warming is leading to major changes in climate patterns with increasing threats to the delivery of sanitation services. Consequently, the need for these services to become more resilient is now urgent.^1,2^ Increasing variability of rainfall and frequency of flooding, in particular, threaten the functioning of sanitation services and increase the risks to public health of poor management of fecal wastes.^2,3^ Increased risks of flooding for sanitation are already resulting from climate change and are projected to increase further as global temperatures increase.^4^ These include pluvial and fluvial flooding,^5^ groundwater flooding,^6^ and coastal flooding.^7^

Climate change is occurring in a global context of slow progress toward achieving universal access to sanitation, with over 1.5 billion people worldwide lacking access to at least basic sanitation.^8^ Of these, 200 million people live in 20 countries most vulnerable to climate change, according to the Global Adaptation Index.^9^ Even without accounting for climate change, inadequate sanitation contributes to an estimated annual 1.4 million deaths and 74 million disability-adjusted life years.^10^

Climate change can impact all parts of the sanitation service chain for sewered and nonsewered services,^2^ but the threats from flooding are of particular concern. Inundation from flooding has the potential to overwhelm or damage sanitation services, leading to widespread fecal contamination in surface and groundwater.^11,12^ Floods may also disrupt supporting services and infrastructure, for example, water supply for flushing and containment emptying services.^13^ Disease outbreaks in the aftermath of floods have been linked to damaged water and sanitation infrastructure and the subsequent contamination of water supplies.^14^ Finally, postcyclone cholera outbreaks have been reported from countries in southern Africa and were linked to damaged sanitation infrastructure.^15^

Flood impacts, like other hazards, disproportionately affect the poor and vulnerable.^16,17^ Repairing or rebuilding toilets comes with a financial burden for households, and those who are reluctant or unable to pay could revert to open defecation which is linked to public health risks and psychosocial stress.^18–20^ Women and girls experience some of the worst impacts when environmental disasters combine with gendered disparities and violence.^21^

In recent years, several resilience frameworks have been developed that propose ideas for improving the resilience of sanitation services and strengthen the response of sanitation infrastructure and services.^1,13,22,23^ Climate-resilient sanitation may be defined as “sewered and nonsewered sanitation systems that can survive, function or recover in the face of climate shocks, stresses or seasonal variability, ensuring that faecal matter is contained along the service chain and does not risk public health”.^22^ Largely, there is consensus that resilience building must take a ‘systems’ approach and go beyond technical interventions to strengthen sanitation policy, institutions and service provision.^24,25^ At the household sanitation facility level, frameworks may also assume certain technological and physical conditions contribute to, or detract from, resilience of the sanitation facility based on commonly held assumptions.^26,27^ While these frameworks offer a strong basis for furthering our knowledge of climate risks to sanitation, more empirical evidence is needed to test assumptions and inform national and subnational planning and investment.

This study aimed to gather evidence of the factors that influence the performance of household sanitation systems that are exposed to flooding in a low- and middle-income-country (LMIC) context. Household sanitation systems, in this study, are those that capture wastewater on-site and are distinguished from sewer-based systems. Focusing on three LMICs—Ethiopia, Uganda, and Nepal—dominant surveys were conducted to understand what variables are correlated with failures of household sanitation facilities due to flooding. In this work, we present the findings and discuss their implications for future climate-resilient sanitation planning and policy. This study is part of the wider Sanitation and Climate: Assessing Resilience and Emissions (SCARE) project focused on the containments and user interfaces of on-site sanitation. Desludging containments were not common across our study sites. Therefore, while we recognize the importance of desludging and treatment services for safely managed sanitation systems, we focused our work on the containments and facilities situated at households. We attempted to capture some data on emptying but found that respondents misunderstood the survey questions. The SCARE project focus was on those forms of on-site sanitation currently used globally to determine their resilience. These are forms of pit latrines and what are often known as ‘septic tanks’ but in reality may be types of holding tanks. Other technologies, such as urine diversion toilets and container-based systems, are promoted, but to date their use is limited.

## 2 Methods

Data were collected from households that own a toilet in selected communities in Nepal, Ethiopia, and Uganda. Data collection for this study was carried out in Ethiopia (November to December 2021), Uganda (February 2022 to December 2023), and Nepal (November 2021 to December 2022) to cover a range of settings and environments that are broadly indicative of where household sanitation systems are found. The dates selected for the surveys ensured that the seasonal pattern in each country was captured to allow our analysis to draw out within-country variation and use this to compare across the three countries. This work focuses on the condition and functionality of household sanitation facilities. Assessment of desludging and treatment services is considered out of the paper’s scope.

### 2.1 Study Areas

The three study areas are described below. Data on flooding were not available in any of the selected study communities, reflecting the generally limited availability of hydrological data in the countries. Each community participating in the study was purposively selected on the basis of the in-country researchers’ prior knowledge and existing evidence from government reports that it was located in a flood-prone area..

#### 2.1.1 Ethiopia

Ethiopia is the second most populous country in Africa, with about 128 million people.^28^ Current coverage of the population with access to at least basic or limited sanitation remains very low (17%) and largely confined to forms on onsite sanitation.^8^ Flood and drought are the top priority hazards in the country based on events’ frequency, area coverage, and number of affected people.^29^ Site selection was purposive and was the closest region to Haramaya University. The sites were not selected to be representative of the country, but the overall range and condition of sanitation were considered to be similar as the national norm. The sites were selected for this study were in Harari Regional State, in the eastern part of Ethiopia. Eleven urban kebeles (the smallest administrative unit in the country) and four rural kebeles were selected: Kebele-1, Kebele-2, Kebele-4, Kebele-5, Kebele-8, Kebele-10, Kebele-12, Kebele-13, Kebele-15, Kebele-16, Kebele-17, Aumer, Gelmeshira, Sofi, and Harawe.

#### 2.1.2 Uganda

Uganda lies across the equator and experiences moderate humid and hot conditions throughout the year.^30^ Just under 40% of the population has access to either basic or limited sanitation, with onsite sanitation predominant.^8^ The country is affected by both floods and droughts, with flood risks more common in the wetter areas located within the Lake Victoria basin, while northern and northeastern districts experience long droughts, which are becoming more frequent.^31^ Two areas were selected for this study: Kampala, which is located along the shore of Lake Victoria, and Gulu, which is in the northern region. These sites were purposively selected, as they were one of the wettest (Kampala) and hottest (Gulu) areas in the country. Sanitation provision across the two sites was similar to, but slightly lower than, the national coverage figures of people with access to improved and unimproved sanitation. Six informal peri-urban settlements spanning six parishes were purposefully selected for this study: Banda, Bwaise III, and Mbuya I in Kampala, and Kirombe, Kasubi, and Tegwana in Gulu.

#### 2.1.3 Nepal

Nepal lies in the foothills of the Himalaya Mountains in Southeast Asia with a population of 30 million.^32^ The country is vulnerable to flooding dangers and natural disasters due to its deep and narrow river basins with frequent mass-wasting events, steep mountain topography, and intense monsoons.^33^ The majority of the population has access to at least basic or limited sanitation (90%), with onsite sanitation predominant.^8^ Site selection was purposive and designed to reflect conditions across the three main ecological zones of the country. Access to improved sanitation across the sites was similar to that at the national level of coverage. This study was conducted in 14 communities of 13 different municipalities: Bethanchowk Municipality, Bharatpur Metropolitan City, Ratnanagar Municipality, Mechinagar Municipality, Solududhkunda Rural Municipality, Pachpokhari Rural Municipality, Dhulikhel Municipality, Jiri Municipality, Waling Municipality, Dhankuta Municipality, Kohalpur Municipality, Mahalaxmi Municipality, and Changu Narayan Municipality. The selection of communities for the study was based on the presence of household sanitation systems, exposure to natural disasters, presence of marginalized communities, and accessibility to the community by the research team. Households in these communities primarily use pit latrines or toilets that flush into holding tanks.

### 2.2 Survey Design

Data were collected using a household survey. Information for the survey was collected by asking a member of the household questions and by observation using a visual inspection form. The survey questions for household members were designed to collect information about socioeconomic characteristics of the respondent, conditions of the household toilet and containment, perceptions of flooding impacts on household sanitation systems, perceptions of climate risks, and household response to the impact of floods on sanitation. The visual inspection form was developed to identify design features, conditions, and locations of the latrines that could potentially make them susceptible to damage or flooding. Both forms were developed from existing frameworks related to sanitation and overall community resilience.^1,22,34–37^ The survey was designed in English, with minimal necessary adjustments made to fit the local context in each country. In Uganda, the questionnaire was piloted in similar informal settlements in each city using both English versions and local languages (Luganda for Kampala or Acholi for Gulu). We found that during the piloting, respondents were happy to answer in English, and therefore, we used this approach in the study. In Nepal, the questionnaire was translated into Nepali, and in Ethiopia it was translated into Amharic and Afan Oromo. In both Nepal and Ethiopia, translation was undertaken by one research team member and back-translated by other members. Any discrepancies were discussed, and consensus was reached on revised wording.

### 2.3 Sample Size Calculation

The sample size for each country was determined using a single population proportion formula. There are no pre-existing data in the three countries or similar countries on what proportion of sanitation facilities are resilient to sanitation. We therefore followed the standard approach in sample size determination in such cases by selecting a *P* of 50% as this maximizes the sample size. We calculated the sample size using a 95% of confidence interval, *Z*, and 5% margin of error, *e*, and allowed for a 5% nonresponse rate to reach the final number of households to be surveyed (Table 1). Pre-existing data on nonresponse rates for the communities participating in this study are not available. Response rates for the Multiple Indicator Cluster Survey (MICS)—a large national-scale survey frequently conducted in low- and middle-income communities—typically has a household response rate of 90–95%.^38^ We therefore selected a 5% nonresponse rate as a reasonable assumption. We estimated the total number of households in each of the study areas using the most recent census data in each country: Nepal: 192,900; Ethiopia: 16,365; Uganda: 24,503.

**Table 1:** Sample Size for the Survey in Each Country

	assumptions				
country	*Z* (95%)	proportion (*P*)	error (e)	nonresponse rate	total sample size
Ethiopia	1.96	0.5	0.05	5%	405
Nepal	1.96	0.5	0.05	8%	418
Uganda	1.96	0.5	0.05	6%	606

A stratified sampling approach was used to select households in each country. In Ethiopia and Uganda, the population was stratified into rural and urban administrative units (kebeles in Ethiopia and parishes in Uganda). In Nepal, the strata were set by ecological zones—Terai, Hills, and Mountains (Table 2). The total sample size for each country (Table 1) was proportionally allocated to each stratum based on its population. Households were selected through systematic random sampling in all three countries. The number of samples allocated to each community within each country was allocated proportional to their relative size, and in each country a sampling interval (K) was selected. In Nepal and Uganda, households were assigned a nominal number, and the first house was selected randomly. A transect through the community was then followed with a K set usually as every fifth house, but in low-density communities in Nepal this was revised to every second house. In Ethiopia, a list of households in each community was obtained from health posts and assigned a number, with K set at 32 for peri-urban areas and 41 for rural areas. The first household in every community was selected randomly, and then every subsequent K household was selected. A total of 1,429 households were included.

**Table 2:** Number of Households in Each Stratum

strata	number of households
Ethiopia (*n* = 405)	
*Urban*	243
*Rural*	162
Nepal (*n* = 418)	
*Mountain region*	90
*Hilly region*	180
*Terai region*	148
Uganda (*n* = 606)	
*Peri-urban informal settlements*	606

### 2.4 Ethical Considerations

Ethical approval for the SCARE project was obtained from the University of Bristol Research Ethics Review Board (approval number 2021-00322-9132). Additional ethical approval was obtained from the Institutional Health Research Ethics Review Committee of Haramaya University (IHRERC) College of Health and Medical Science (approval number IHRERC/192/2021) and the Institutional Research Committee at Kathmandu University School of Medical Science (approval number 15/2021). Due to the aftereffects of COVID-19 restrictions at Kyambogo University, the University of Bristol ethics approval was permitted by Kyambogo University to cover data collection in Uganda. In each country, the study participants were briefed about the purpose of the study, and their informed written consent was collected and stored in the KOBO survey toolbox before the survey.

### 2.5 Data Collection

The survey and visual inspection forms were uploaded to the Kobo toolbox and piloted in a subset of households in the three countries before being administered in the communities. Enumerators were recruited and trained in the use of the Kobo toolbox.

### 2.6 Data Analysis

To obtain a sufficiently large sample size to draw meaningful conclusions, our analyses were conducted by using the combined data from all three countries. Data analysis was undertaken using STATA 18 ^39^ to identify associations between household sanitation system characteristics, household characteristics, and sanitation management practices with flood-related impacts on household sanitation systems. This initial analysis was carried out for all communities surveyed by using a Pearson correlation.

Associations between predictor variables (Table 3) and negative outcomes related to toilets (inundation of the toilet, inability to flush the toilet, and overflow of the containment unit) were explored by using a logistic regression model (eq 1).

1 $$\ln\frac{p\left(X\right)}{1-p\left(X\right)}=\beta_{0}+\beta_{1}X_{1}+...+\beta_{p}X_{p}$$

where the log of odds, $\ln\frac{p\left(X\right)}{1-p\left(X\right)}$ is the probability of being negatively affected by a flood, and *X*_1_, ... ,*X*_*p*_ are the predictors variables with their coefficients β_1_, ···, *β*_*p*_. As Table 3 shows, the predictors considered in the model are related to (i) the socioeconomic characteristics of the households (education, age, employment), (ii) the characteristics of their toilets (cracks in latrines, leaks in superstructure roof, etc.), and (iii) the geographical aspects (rural-urban or ecological zone). Only significant variables were considered in the final model. Finally, different tests were conducted (goodness-of-fit, multicollinearity, sensitivity, specificity, etc.) to validate the accuracy of the model (Supporting Information).

**Table 3:** Household and Sanitation Characteristics of Households Included in the Logistic Regression Model

variable	count (%)
Gender of Respondent	
Male	405 (55.2%)
Female	329 (44.8%)
Type of Employment of Respondent	
Not formally employed (e.g., farmer, student, unemployed, homemaker, etc.)	264 (36.4%)
Formally employed (e.g., government employee, private employment, etc.)	462 (63.6%)
Education of Respondent	
Completed secondary/high school level or higher	360 (50.1%)
Primary level education or less	359 (49.9%)
Respondent Familiar with the Term “Climate Change”[Table-fn t3fn10]	
Yes	428 (58.2%)
No	308 (41.8%)
Fully Sealed Containment Unit	
Yes	75 (10.3%)
No	655 (89.7%)
Owner Has Paid to Make Latrine More Resilient	
Yes	169 (23.6%)
No	548 (76.4%)
Proximity of Latrine to Flood-Prone Water Body	
Yes	228 (31.1%)
No	505 (68.9%)
Toilet Is Raised above the Ground	
Yes	415 (56.7%)
No	317 (43.3%)
Presence of Cracks in Latrine Slab	
Yes	101 (13.8%)
No	630 (86.2%)
Presence of Leaks in Superstructure Roof	
Yes	204 (27.8%)
No	529 (72.2%)
Presence of Permanent Latrine Superstructure	
Yes	563 (77.4%)
No	164 (22.6%)
Location of Household	
Peri-urban or urban	620 (84.0%)
Rural	118 (16.0%)
Number of households in Ethiopia	215 (29.1%)
Number of households in Uganda	309 (41.9%)
Number of households in Nepal	214 (29.0%)
Number of households in Terai region of Nepal	120 (16.3%)
Variable	Mean
Mean age of respondent	41
Mean number of users per toilet	12

a In Ethiopia, the terms Ye-Ayer Nibret Lewit (Amharic) and Jijjirama qilleensaa (Afan Oromo) were used, and in Nepal, the Nepali word Jalvayu Pariwartan was used. In Uganda, the questionnaire was delivered in English, and this was used to explain climate change.

The analysis of predictor variables in the logistic regression model was initially for only those communities where more than 20% of households reported exposure to flooding to exclude communities where flooding is not significant from the model. A threshold of 20% was initially selected because the research team considered that this would be a level of exposure where damage would be sufficiently substantial that action would be expected to be taken. The team judged that fewer than 20% of households reporting exposure to flooding would indicate that the community did not experience significant flooding. We repeated the analysis using thresholds of households reporting exposure to floods set to 10% and 30% to understand what influence the threshold level has in terms of reported flood damage. It should be noted that these thresholds were arbitrary but reflect the experience of the researchers that such thresholds are of value for policy-makers in identifying priority areas. Exposure to flooding was determined through the proportion of households within a community that answered yes to the question “Is the community/surrounding area affected by rising groundwater table, storm surges, river flooding or flash flooding from seasonal channels?”

This analysis was carried out to identify associations between household sanitation system characteristics, household characteristics, and sanitation management practices, with households reporting practicing open defecation following flood damage to the sanitation system. The same logistic regression method was again applied to all households that reported that they had ever experienced damage to their sanitation facility due to flooding (regardless of the proportion of their community reporting exposure to flooding) to determine predictor variables associated with the practice of open defecation following flood damage to the sanitation facility.

## 3 Results

### 3.1 Exposure to Flooding and Negative Outcomes

The proportion of households within a community that reported experiencing a negative outcome was positively correlated with the proportion of households exposed to flooding (*p* < 0.01; *R* ^2^ = 0.29). Experiencing a negative outcome due to flooding was determined by the proportion of households in a community that reported that they have ever experienced inundation of the toilet, an inability to flush the toilet due to heavy rainfall or flooding, or overflow of their containment unit due to heavy rainfall and flooding. Across the entire sample, more than half (*n* = 810, 57%) of households reported exposure to flooding, and 264 households (18%) reported experiencing at least one of the three negative outcomes related to flooding. Figure 1 shows a scatterplot of community exposure to flooding against experiences of a negative outcome associated with flooding.

**Figure 1:**
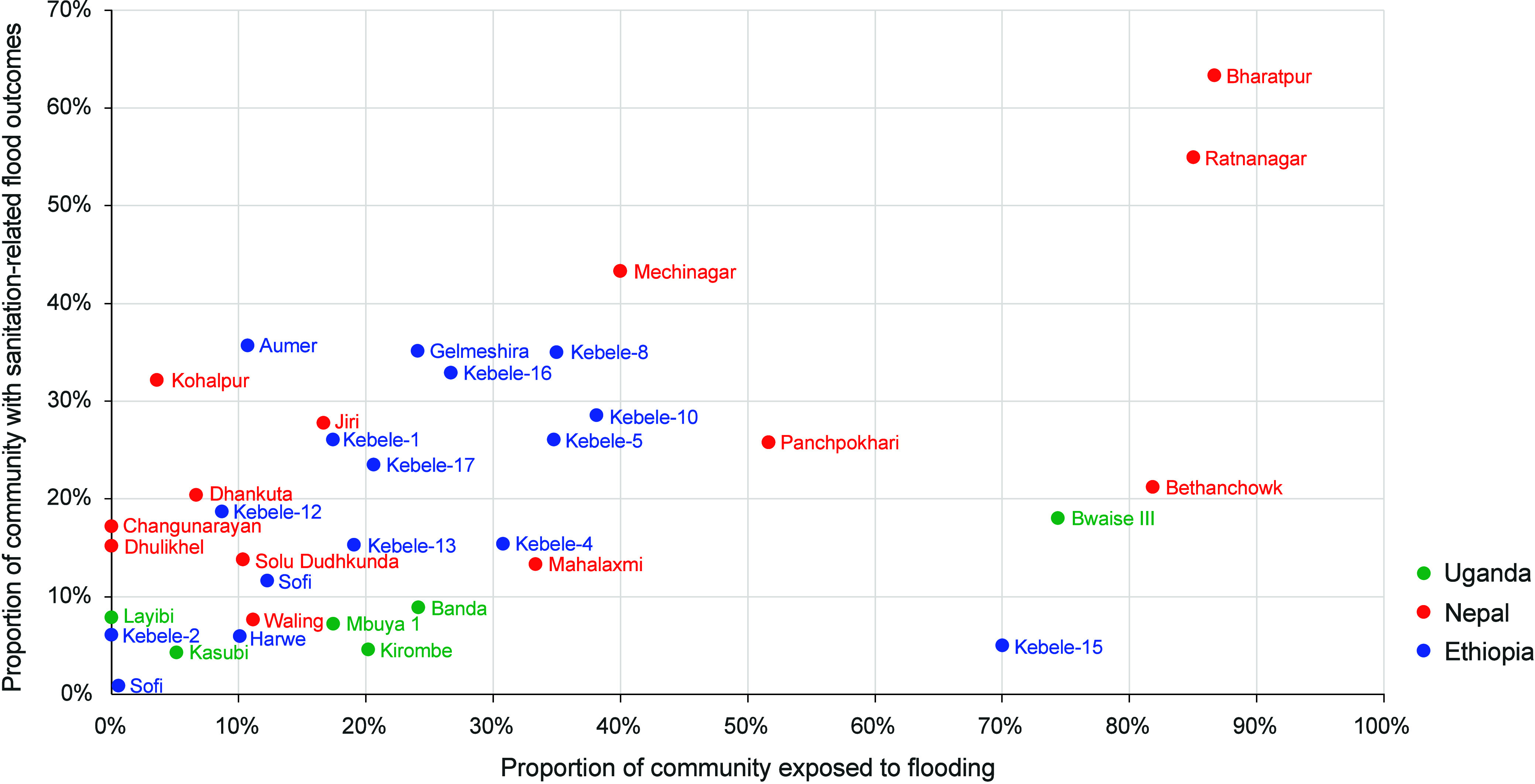
Proportion of studied communities reporting exposure to flooding- and sanitation-related flood outcomes.

We then used logistic regression to analyze the importance of predictor variables. Out of the 35 communities included in the total sample, 18 met the initial criteria of 20% or more of households in the community that reported exposure to flooding. These 18 communities included 738 households participating in the study. Among the 738 households included in the model, 322 (43.6%) reported being exposed to flooding, and 176 (24%) reported that they experienced a negative outcome for their sanitation system associated with flooding.

### 3.2 Characteristics of Respondents and Household Toilets

The characteristics of the respondents and their toilets are typical of those in an LMIC. Table 3 shows the results of the descriptive analysis of the predictor variables for the 738 households included in the logistic regression model when the threshold for flood exposure was set to 20%. Most (84%) households were in peri-urban or urban areas. Half (50.1%) of the respondents had completed secondary or high school, and respondents were roughly equally represented by women (44.8%) and men (55.2%). A majority (58.2%) of respondents reported being familiar with the term “climate change” (local terms for climate change that are well-understood were used to describe climate change), but only about a quarter (23.6%) reported making a payment to make their latrine more resilient to climate hazards. Regarding latrine quality, a significant number of respondents reported cracks in their latrine slab (13.8%) or leaks in the latrine superstructure (27.8%).

Overall, Table 3 suggests that many sanitation facilities included in the logistic regression model had characteristics (e.g., exposure to flooding, unsealed containment, damaged or impermanent infrastructure) that would make them susceptible to disruptions from flooding.

### 3.3 Variables Associated with Negative Outcomes

Among the communities included in the model, three variables were found to be significantly positively correlated with negative outcomes: the presence of cracks in the latrine slab that might allow water to enter the containment (*p* < 0.001), an impermanent latrine superstructure (*p* = 0.011), and households located in the Terai region (lowlands) of Nepal (*p* < 0.001) (Table 4). An impermanent latrine superstructure was defined as one built without concrete, bricks and mortar, or other sturdy materials. Often, impermanent latrines are built with mud and stone, tarpaulin, or sheet metal that is not securely fastened.

**Table 4:** Logistic Regression Model Results for Flood-Related Outcomes in Household Sanitation Facilities

variables	odds ratio	standard error	*z*	*P*-value^a^
Age	1.02	0.01	1.39	0.165
Gender of respondent	1.43	0.41	1.25	0.211
Education of respondent	1.17	0.26	0.71	0.480
Type of employment of respondent	0.93	0.21	–0.33	0.740
Proximity of latrine to flood-prone water body	3.36	0.55	7.46	0***
Presence of cracks in latrine slab	6.59	2.03	6.13	0***
Presence of permanent latrine superstructure	0.42	0.14	–2.55	0.011**
Household located in Terai region	5.31	1.06	8.41	0***
Constant	0.07	0.04	–4.21	0***

a ***p < 0.01, **p < 0.05.

Household demographics, familiarity with the term ‘climate change’, whether the latrine was raised above the ground, and other infrastructure characteristics were not significantly associated with any negative outcome.

Changes occurred when the threshold for inclusion in the sampling was adjusted. When the logistic regression model was run to include communities in which 30% or more of households reported exposure to flooding, latrines proximal to flood-prone water bodies, cracks in the latrine slab, and households located in the Terai region were still significant variables, as they were with a 20% threshold. Additionally, a fully sealed containment unit and households in peri-urban or urban areas became significant variables. However, a permanent latrine superstructure was not as significant as it was at a 20% threshold. When the model was run with a threshold of 10%, the results were the same as with a 20% threshold, except the presence of leaks in the superstructure roof became significant, and the presence of a permanent latrine superstructure was not significant (Table 5).

**Table 5:** Variables Correlated with Flood-Related Outcomes for Household Sanitation Facilities Resulting from Logistic Regression Models with Three Different Inclusion Criteria

Communities with 10% of households reporting flood exposure	Communities with 20% of households reporting flood exposure	Communities with 30% of households reporting flood exposure
Proximity of latrine to flood-prone water body (*p* < 0.01)	Proximity of latrine to flood-prone water body (*p* < 0.01)	Proximity of latrine to flood-prone water body (*p* < 0.01)
Presence of cracks in latrine slab (*p* < 0.01)	Presence of cracks in latrine slab (*p* < 0.01)	Presence of cracks in latrine slab (*p* < 0.01)
Household located in Terai region (*p* < 0.01)	Household located in Terai region (*p* < 0.01)	Household located in Terai region (*p* < 0.01)
Presence of leaks in superstructure roof (*p* < 0.01)		
	Presence of permanent latrine superstructure (*p* = 0.011)	
		Fully sealed containment unit (*p* = 0.52)
		Household located in peri-urban or urban area (*p* = 0.044)

### 3.4 Open Defecation Due to Flood Damage

Among all households across the three countries, 72 reported that family members sometimes practiced open defecation due to flood damage to their sanitation system. Table 6 shows logistic regression model results for open defecation following flood damage to latrines for all households participating in the study (irrespective of the proportion of their community reporting exposure to flooding). Constructing a new toilet, repairing the toilet, using an alternative toilet (such as a neighbor’s toilet), or continuing to use the damaged toilet were also frequent responses. The presence of cracks in the latrine slab (*p* = 0.022), being in a rural area (*p* < 0.001), and discomfort with using a neighbor’s latrine (*p* = 0.012) were most positively correlated with open defecation following flood damage. Household demographics were not correlated with open defecation.

**Table 6:** Logistic Regression Model Results for Open Defecation Following Flood Damage to Latrines[Table-fn t6fn100]

variables	odds ratio	standard error	z	*P*-value^a^
Age	1.01	0.01	1.12	0.261
Gender of respondent	1.33	0.44	0.87	0.386
Education of respondent	0.50	0.18	–1.91	0.056*
Type of employment of respondent	1.52	0.42	1.51	0.130
Respondent familiar with the term “climate change”	0.35	0.20	–1.85	0.065*
Presence of cracks in latrine slab	2.70	1.18	2.28	0.022**
Household located in peri-urban or urban area	0.25	0.09	–3.87	0***
Containment eventually requires emptying[Table-fn t6fn100]	3.39	2.20	1.89	0.059*
Respondent comfortable using neighbor’s latrine	0.09	0.09	–2.52	0.012**
Constant	0.09	0.07	–2.81	0.005***

a Respondents were asked if their toilets flooded or were damaged in the rainy season. If they responded yes, then they were asked what type of repair was needed. One of the options to this multiple-choice question was ‘empty containment.’

b ***p < 0.01, **p < 0.05, *p < 0.1.

## 4 Discussion

This section explores the study’s key findings and situates them in the context of other related literature: (i) the correlation between flood exposure and flood-related outcomes for sanitation facilities; (ii) the association between the quality sanitation systems’ construction and flood-related outcomes; (iii) variables not found to be associated with flood-related outcomes; and (iv) variables associated with the practice of open defecation following flood damage. The discussion provides potential explanations for each finding and offers subsequent recommendations for sanitation planning and policy in the studied sites. Finally, the discussion reflects on the use of thresholds for defining flood-exposed communities and approaches to minimize reversion open defecation and provide backup access to other sanitation facilities.

### 4.1 Settlements Exposed to Flooding

The results provide evidence that household sanitation systems in the study sites in Ethiopia, Uganda, and Nepal are sensitive to flooding. Communities with higher proportions of households reporting flooding also experienced higher rates of sanitation system failures. This aligns with frequent assertions in the literature and sanitation discourse that flooding significantly impacts household sanitation systems.^1,24,27,40^ Therefore, consideration of the exposure of settlements to flooding when designing and implementing household sanitation interventions is warranted.

The Terai region of Nepal exemplifies an area highly exposed to flooding due to its geographical and topographical characteristics. Situated at the base of the Himalayas, the region experiences prolonged flooding during the monsoon season as rainwater from the mountains drains into this low-lying area.^41^ Additionally, changes in land use, engineering works, landslides, and increasing rainfall intensity exacerbate flooding in the region.^41^ These factors likely explain why our model showed the Terai region to be significantly correlated with flood outcomes. However, mountain areas in Nepal are also vulnerable^42^ and should not be neglected in policy.

In Uganda and Ethiopia, urban planning challenges may significantly contribute to flood exposure. In Uganda, low-income households in informal settlements are often located in flood-prone areas such as wetlands and hilly slopes, which are also susceptible to landslides and lack access to essential urban services.^43^ The expansion of these informal settlements into vulnerable areas and their encroachment on wetlands exacerbate flood risks. Similarly, in Ethiopia, rapid population growth, poor urban planning, and the loss of green infrastructure that could mitigate peak flows have driven the expansion of informal settlements, increasing community exposure to flooding.^44^

The ‘double exposure’ problem^45^—increasing exposure to flooding driven simultaneously by climate change and inequitable economic development that leads to the formation of informal settlements in flood-prone areas—is difficult to counteract. Enforcement of regulations to discourage construction in flood-prone areas and protection of wetlands should be pursued,^46^ but governments in LMICs have historically been challenged to accomplish this. The management of flooding in urban areas, often through improved drainage or nature-based solutions, is the subject of much discussion.^47–49^ Such initiatives would likely provide cobenefits for sanitation systems in the studied sites in Uganda and Ethiopia. Nonetheless, sanitation planners and implementers will need to overcome challenges of flood exposure in the near-term. In communities that are already routinely exposed to flooding, future research should consider the relative costs and merits of controlling flooding (e.g., through improved drainage), resisting or accommodating the effects of flooding through redesign of sanitation facilities (e.g., raised infrastructure), and relocating critical infrastructure.

### 4.2 Quality of Construction and Maintenance

The findings emphasize the importance of construction quality and maintenance in ensuring the resistance of latrines to flooding in the studied sites, similar to the findings of previous studies in Africa.^27^ The positive correlation among the presence of cracks in the latrine slab, impermanent superstructures, and reports of negative outcomes for toilets highlights the need for durable materials and proper construction practices. These findings align with other empirical studies in LMICs, which have shown that latrines constructed by nonexperts are more prone to flood damage than those built by trained masons.^50^ Similarly, unimproved sanitation systems are more likely to experience flood-related problems than improved systems.^13^ However, simply sealing the slab or reinforcing the superstructure is not sufficient to make a latrine flood-resilient. Instead, as other studies suggest, the use of high-quality construction materials and techniques is essential to minimize the risk of flood-induced failures.^27,51^

These findings are consistent with the broader literature on rural sanitation development. A common challenge in demand-based sanitation approaches, such as Community-Led Total Sanitation, often employed in rural LMICs, is the construction of substandard latrines by households.^52^ The poor quality of these latrines often contributes to their failure, leading users to revert to open defecation.^53,54^ Consequently, there is a strong case for promoting durable sanitation technologies in these settings.^52–54^ Promoting high-quality latrines is beneficial in both flood-prone and nonflood-exposed contexts and should be recommended broadly.

In all three countries, the affordability of good-quality building materials for low-income households may be a constraint. Anecdotal evidence from rural areas of Ethiopia and Nepal suggests that the use of poor-quality materials may also be a consequence of limited access to good quality sanitation products. In informal settlements of Uganda, limited space and financial constraints can prevent households from constructing more permanent sanitation facilities.^55^ Addressing these issues requires improving access to high-quality sanitation products and raising community awareness of the importance of good-quality sanitation facilities. Strategies to achieve this could include enhanced sanitation marketing, the provision of subsidized sanitation products, the establishment of minimum standards for sanitation facilities and provision of training to local sanitation facility builders, and enforcement of relevant policies.^52^ Moreover, standards for high-quality sanitation products and installations should be particularly stringent in areas with a high risk of flooding. Potential initiatives to improve the affordability of high-quality latrines include mobilizing villages funds, establishing local entrepreneurs to make robust materials locally available, targeted subsidies for the lowest-income households, and purchasing materials in bulk to obtain discounts.^56^ Policies that support the professionalization of sanitation services, including in rural areas, may help to achieve these outcomes.

### 4.3 Raising Toilets, Toilet Characteristics, and Household Demographics Require Further Investigation

Although this study did not find a correlation between negative flood-related outcomes and other variables often posited in sanitation literature as influential—such as raising the toilet,^27^ fully sealing the containment unit,^23^ and household demographics^25^—these factors may still be significant in some contexts.

This study explored whether raising the toilet above ground was associated with better performance outcomes but did not examine the elevation of other parts of the sanitation system. Our finding that raising the latrine was not a significant variable contrasts with a previous study that asserted latrine-raising is an effective solution to flooding.^57^ In possible scenarios where floodwater ingress into the containment unit (e.g., through outlets or access hatches) is the primary cause of failure, raising the toilet alone will have little impact. Furthermore, other studies have noted that raised latrines may encourage ‘flooding out’ of pits, where fecal sludge is intentionally released in the environment through a drain installed in an elevated portion of the pit.^58^

Thus, it is critical to understand the specific mechanisms by which flooding affects sanitation system performance to determine whether and which components of the system should be raised. It is also difficult to ascertain the optimal height to which sanitation infrastructure should be elevated, especially in contexts lacking reliable flood-level data or where flood intensity is changing over time. Further observational research is needed to explore these variables in greater depth to identify conditions under which they significantly improve sanitation outcomes during floods. Nature-based solutions to flooding that reduce the depth and energy within flood events also warrant investigation, as these may increase resilience of sanitation through reducing exposure.

Other variables that were not found to be significant in this study also warrant further investigation. Household demographics, including the gender, education, and employment status of the head of household, were also not found to be significant variables in our model. Gender and education, however, had odds ratios, indicating some effect. Employment status is only a rough proxy for household income and wealth and may not fully capture the effects of financial well-being (or lack thereof). Nevertheless, social and economic dimensions are likely to influence a household’s ability to effectively manage the impacts of flooding on sanitation access.^25^ An association between household demographics and susceptibility to harm from climate hazards and disasters is well-established.^59–61^ Although this association is understudied in the context of sanitation, other site-specific studies found that lower-income households are more likely to abandon toilets after flood damage,^62^ latrine rebuilding following effects of heavy rainfall was associated with the education level of community members,^63^ and latrine superstructure flooding is associated with household income.^64^

Qualitative assessments tailored to specific country contexts could provide deeper insights into these dimensions and guide future resilience-building efforts. Finally, we aimed to consider containment-desludging in our model but were unable to collect adequate data on this because many respondents misunderstood the survey questions. Future research should consider the effects of desludging practices on flood-related outcomes for sanitation systems. Future research could also consider aspects such as groundwater level, soil type, and evidence of repairs to facilities. However, to provide meaningful results, methods other than household surveys would be required to collect reliable data and in the case of groundwater level a campaign of measurement to reflect seasonal variation.

### 4.4 Defining Thresholds for Identifying Flood-Exposed Communities

In this study, the choice of a flood exposure threshold for determining which communities to include in the logistic regression model was significant. Adjusting the inclusion criterion, setting the proportion of households reporting exposure to flooding at 10%, 20%, or 30%, produced similar yet distinct results regarding variables positively correlated to flood-related sanitation outcomes.

This finding has implications for policymakers. To effectively target support to flood-exposed communities, policymakers must first define what qualifies a community as “flood-exposed”. They then identify those communities and determine the appropriate forms of support. Our study suggests that the criteria used to define flood exposure can influence subsequent data analysis on community needs and, in turn, shape policy responses. This is equally true if a different method from household surveys (e.g., environmental assessments) is used to assess flood exposure. Because policymakers typically operate at the community level (or higher), setting inclusion criteria at the community level or on a broader scale is reasonable. However, further research is needed to establish the most appropriate threshold for defining flood exposure.

Despite variations in significant variables when the flood exposure threshold is adjusted, two key factors remain consistent: infrastructure quality and geography. The presence of cracks in the latrine slab is a significant variable across all three threshold levels, and the quality of the latrine superstructure is significant in two of three cases. The lack of a fully sealed containment unit does not necessarily indicate poor quality. Some units are designed to infiltrate liquids into the surrounding soil, which can be safe given an adequate distance from water sources,^6^ but it may also reflect lower-quality sanitation systems. The remaining significant variables across the three threshold levels relate to the geographical characteristics of the sanitation facility’s location. Together, these findings reinforce the importance of a high-quality infrastructure and highlight the need for special attention to particularly challenging environments.

### 4.5 Reversion to Open Defecation

This study’s finding that some households practice open defecation due to flooding impacts aligns with existing literature, which has found the same outcome in other contexts.^13,19,63^ The increasing frequency and severity of flooding events driven by climate change and other environmental factors present a significant challenge to the progress made in achieving safely managed sanitation. Such events risk undermining these gains, slowing progress, or even reversing advancements in sanitation access.

Our finding that open defecation is associated with discomfort in using a neighbor’s toilet is consistent with the CLTS literature on slippage and the role of social capital in sustaining sanitation access.^63,65^ This includes instances following flood damage.^19^ We did not include specific questions in the survey to explore why people felt uncomfortable about using a neighbor’s toilet, but several factors may contribute to this discomfort: individuals may feel ashamed or embarrassed to ask a neighbor for access, or they may perceive using a neighbor’s toilet as insufficiently private or safe.

The provision of publicly accessible communal or institutional sanitation facilities can act as a vital backup for households whose toilets are damaged by floods. These facilities are particularly effective in densely populated areas, where they can serve a large number of people. However, communal facilities, such as those operated by local councils, NGOs, or institutions, often face challenges including poor hygiene compared to neighbor-shared toilets.^66^ It is essential to address the challenges associated with communal facilities, including poor management, limited functionality, public reluctance to use them, and difficulties in maintaining cleanliness, as highlighted in previous studies.^67,68^ Further, communal facilities should be strategically located, well-designed, and effectively managed to withstand the impacts of flooding. Public policies can help guide appropriate management models for communal facilities that serve as vital backups for community members.

Open defecation may also reflect limited alternatives and challenges in maintaining a sanitation infrastructure. The association between open defecation and rural settings likely reflects the lack of sanitation alternatives and limited access to markets for repairs in these areas. Additionally, the observed link between cracks in the latrine slab and open defecation suggests that latrine quality and household investment in maintenance play key roles in sustaining sanitation access. To address these challenges, as discussed above, promoting the construction of high-quality, durable latrines is crucial. This may involve investments in establishing sanitation markets in flood-prone areas^62^ and providing subsidies for flood-resistant sanitation products.^69^

### 4.6 Limitations

There are limitations to this study. First, not all negative sanitation outcomes, such as the inability to flush toilets or the overflow of containment units, can be attributed to flooding. We did not collect data on the length of time facilities were damaged before repair or whether reversion to open defecation was permanent or temporary. Both of these aspects would be useful to explore in future studies. Although households reported these issues in conjunction with flood events, other factors, such as poor maintenance, could also contribute to these outcomes. This creates some uncertainty in establishing a direct causal link between flooding and specific sanitation failures. Next, the study relied on self-reported data to determine whether households had sealed containment units. Given that containment infrastructure is underground and usually not directly accessible, the research team could not verify the condition of the containment units, and respondents may have had limited knowledge about their condition. This uncertainty could affect the accuracy of responses and, consequently, the interpretation of results regarding the containment effectiveness during floods. The study found that respondents did not consistently interpret the questions about emptying containments in the same way. This meant that we could not examine whether such behaviors influence resilience.

Another limitation is the selection of the study sites. While the purposive selection of communities ensured representation of different geographic and climatic contexts, it may limit the generalizability of findings beyond the study areas. Communities with different governance structures, infrastructure investment levels, or flood adaptation measures may experience different sanitation-related impacts.

Finally, exposure to flooding was measured subjectively through self-reporting by households because hydrological and flooding data were scarce in the study settings. Households may have had different understandings of the term “flooding”, which could have affected their responses and, consequently, whether they were included in the logistic regression model. Households were asked to consider flooding from a rising groundwater table, storm surges, river flooding, or seasonal flooding collectively, and lumping these different together limits a more nuanced understanding of the types of flooding that users experience. The analyses were conducted for communities in which at least 10% of households reported exposure to flooding to increase the likelihood that communities included in the analyses were experiencing at least some meaningful level of flooding.

## 5 Conclusions

This study provided empirical evidence of factors that influence the resilience of household sanitation facilities in Uganda, Ethiopia, and Nepal to flooding. In particular, it examined variables that correlated with sanitation system failure when flooding is experienced and found that the quality of household sanitation infrastructure and geographical aspects emerged as most important among other considered variables. The study also found that the quality of infrastructure was correlated with the practice of open defecation following flood damage to latrines, along with being in a rural area and discomfort using a neighbor’s latrine. Together, these findings point to the need to make good quality sanitation products and designs readily accessible to communities, especially in geographically challenging environments.

Further research would strengthen the evidence base for informing policy makers about supporting climate resilient sanitation. Research on other variables, such as desludging frequency and raising of the containment unit, could shed light on other characteristics that improve performance when flooding is experienced. Future research could also gather empirical evidence of factors supporting other stages of the sanitation chain, such as desludging and treatment services, to operate effectively under difficult environmental conditions. Beyond flooding, other hazards, such as water shortages due to dry spells and windstorms, can also severely disrupt sanitation services and warrant similar research. Finally, enhancing the resilience of sanitation systems must go beyond technical interventions and extend to strengthening crucial support systems, such as those related to sustainable financing, regulations, and monitoring and evaluation. Further research should support innovations in supporting systems and ensuring interventions provide equitable benefits.

As the climate crisis continues to heighten, sanitation stakeholders must act with urgency while maintaining a reasoned and evidence-based approach. Establishing evidence based on what makes sanitation systems more resilient and acting on it through policy and practice should be a priority for all governments and their supporting partners.

## Supplementary Material



## Data Availability

Due to the sensitivity of the data involved, these data are published as a controlled data set at the University of Bristol Research Data Repository data.bris at 10.5523/bris.1o1bcoz1ollwu2nqu6ys7cp5ul. The metadata record published openly by the repository at this location clearly states how data can be accessed by bona fide researchers. Requests for access will be considered by the University of Bristol Data Access Committee, who will assess the motives of potential data reusers before deciding to grant access to the data. No authentic request for access will be refused, and reusers will not be charged for any part of this process.

## Notes

1) The survey questionnaire used in data collection; 2) Table S1 provides details of households and sanitation characteristics surveyed in each country (PDF)
