# Supplementary material for: What Makes Household Sanitation Systems Resilient to Floods? Evidence from Ethiopia, Uganda, and Nepal
Source: ACS ES T Water. 2026 Feb 17;6(3):1531–42. doi: 10.1021/acsestwater.5c01055 (PMC12993853; doi:10.1021/acsestwater.5c01055)
Supplement: Supplementary file 1 [file ew5c01055_si_001.pdf]

What makes household sanitation systems resilient to floods? Evidence from Ethiopia, Uganda, and Nepal Supplementary material

Jeremy Kohlitz1, Abraham Geremew2, Kenan Okurut3, Prativa Poudel4, Anish Ghimire4,5, Anisha Nijhawan6, Alejandro Valenzuela1, Jay Falletta1, Anjali Manandhar-Sherpa6, Juliet Willetts1, and Guy Howard6*

1 Institute of Sustainable Futures, University of Technology Sydney, Australia

2 Department of Environmental Health, College of Health and Medical Sciences, Haramaya University, Ethiopia

3 Kyambogo University, Uganda

4 Aquatic Ecology Centre, Kathmandu University, Nepal

5 Asian Institute of Technology, Thailand

6 School of Civil, Aerospace and Design Engineering and Cabot Institute for the Environment, University of Bristol, Bristol BS8 1TR, UK

*Corresponding author; email: guy.howard@bristol.ac.uk

Survey Questionnaire

**Part I. Household Information**	
Municipality :	Ward No.: .... Tole: .... GPS: ....N.............................. E
Respondent's Name: ……………………………………	Contact Number: ....
Respondent's Age:	Respondent's Sex: Female............Male.............Other........
Head of the family's Name:	Head of the family's Age: ....
Head of the family's Sex:	Relation between Respondent & Head of the family: ....
Total No. of family member:	

**<u>Part 2 : Socio-Economic Status</u>**				
S.N.	Socio-economic status			Remarks
1	Respondent's educational qualification	Doctorate (PhD or above)	9	
		Graduate	8	
		Undergraduate	7	
		Intermediate /Diploma	6	
		High school certificate	5	
		Middle school certificate	4	
		Primary school certificate	3	
		Literate (able to read and write)	2	
		Illiterate	1	
2	Head of the family's educational qualification	Doctorate (PhD or above)	9	
		Graduate	8	
		Undergraduate	7	
		Intermediate /Diploma	6	
		High school certificate	5	
		Middle school certificate	4	
		Primary school certificate	3	
		Literate (able to read and write)	2	

		Illiterate	1	
2.	Head of the family's occupation	Job holder	11	
		Entrepreneur	10	
		Skilled Worker (plumbers, mechanics)	9	
		Shop & Market Sales Workers	8	
		Agricultural/ farming (own land)	7	
		Agriculture/ farming (other's land)	6	
		Livestock farming	5	
		Craft & Related Trade Workers	4	
		Student	3	
		Daily wage worker	2	
		Unemployed	1	
3.	Toilet Type	1. Single pit latrine<br>2. Double pit latrine<br>3. Septic Tank (watertight, baffle wall, two-chamber, outlet)<br>4. Holding Tank (single chamber, fully lined/sealed)<br>5. Holding Tank(single chamber, partially lined/unsealed)<br>6. Biogas connected toilet<br>1. ECOSAN<br>2. Others ……………..<br><br>(Please specify)		
4	Number of Toilet users			
5	Total number and location of the toilet	1. Inside the house<br>2. Outside the house<br><br>Number of toilets: ……….		
6	Location of containment	1. Under the house plinth<br>2. Outside in the compound		
7	Age of the containment	1. Less than 1 year<br>2. 1-2 years<br>3. 3-5 years<br>4. 6-10 years<br>5. More than 10 years		
8.	Size of containment (m^3)	……..length…….breadth……height		

9	What is the source of water used for the latrine?	1. Private tap<br>2. Public tap<br>3. Pond<br>4. River<br>5. Water tanker<br>6. Well<br>7. Deep boring<br>8. Hand pump/ tubewell<br>9. Rainwater<br>10. Other …………..	
10	How long does it take to fetch the water?	1. Less than 5 min<br>2. 6-10 min<br>3. 11-15 min<br>4. 16-20 min<br>5. 21-25 min<br>6. 26-30 min<br>7. More than 30 min	

**Part II: User's survey**			**Remark**
**I. Questions on risks in the wider environment**			
1	Is the community/surrounding area affected by rising groundwater table, storm surges, river flooding or flash flooding from seasonal channels?<br><br>If "No" then go to Q.N. 2	1. Yes<br>2. No	
	**II: Questions on risks at the latrine**		
2	If the response to Q.N. 1 is "No" then skip this question.<br><br>Does the latrine superstructure get affected during dry or monsoon season?<br><br>If the response is "No" then go to Q.N. 3	1. Yes<br>2. No	
3	Can you physically access and use the latrine during the rainy season?	1. Yes<br>2. No	
4	Does the containment (septic tank, pit) get damaged or flood during the rainy season?<br><br>If the response is "No" then go to Q.N. 6	1. Yes<br>2. No	
5	If the response is "Yes" in Q.N. 4, then specify the reason	1. Rising groundwater table<br>2. Flood water inundation<br>3. Heavy rainfall	

		4. Landslide<br>5. Others<br>……………<br><br>(Please specify)	
6	Does the latrine stop flushing or spill faeces from backflow during wet weather?	1. Yes<br>2. No	
7	Does the latrine get blocked during dry weather?	1. Yes<br>2. No	
	If the response to Q.N. 2-7 is "No" then go to Q.N. 14		
8	If your responses to Q.N. 2-7 is "Yes", how long did it take for the latrine to be repaired (number of days)?	________(days)	
9	What were the repair works done?	1. Rebuild the superstructure<br>2. Plaster the toilet floor<br>3. Change the toilet roof<br>4. Plaster the containment<br>5. Rebuild containment<br>6. Empty containment<br>7. Others (Please specify)<br>…………	
10	Were you able to use the latrine during this time?	1. Yes<br>2. No	
11	What did you do when the latrine was damaged?	1. Used alternate toilet<br>2. Used neighbor's toilet/latrine<br>3. Defecate in the open<br>4. Other …………<br><br>Please specify	
12	Would you feel comfortable asking your neighbors or friends for help with repairing your latrine?	1. Yes<br>2. No	
13	Would you feel comfortable using your neighbors' or friends' facilities in case your latrine is inaccessible?	1. Yes<br>2. No	

14	Are odors from the latrine worse during a particular season?	1. Yes<br>2. No	
15	If the answer is Q.N. 14 is "Yes" then on which season this particular odor occurred?	1. Spring (Mar-May)<br>2. Monsoon/ Summer (Jun-Aug)<br>3. Autumn (Sep-Oct)<br>4. Winter (Nov-Feb)	
16	**For water-based toilets**: How frequently do you have inadequate quantities of water to flush the toilet during prolonged dry seasons?<br><br>If the response in Q.N. 4 of section 2 is "ECOSAN", skip this question.<br><br>If the response is "Never" then go to Q.N. 18	1. Multiple times per week<br>2. Multiple times per month<br>3. Once per month<br>4. Less than once per month<br>5. Never	
17	If there's water scarcity, what time of the year do you experience it the most?	1. Spring (Mar-May)<br>2. Monsoon/ summer(Jun-Aug)<br>3. Autumn (Sep-Oct)<br>4. Winter (Nov-Feb)	
18	Does the containment unit have an outlet?<br>If the response is "No" then go to Q.N. 20	1. Yes<br>2. No	
19	If the answer is Q.N. 18 is "Yes", where is the outlet connected to?	1. Open area<br>2. Agricultural area/ field<br>3. A drain or channel<br>4. Others ……………<br>(Please specify)	
**III. Questions on risks to the supply chain**			
20	Is the containment accessible for regular pumping or emptying?<br>If the response is "Yes" then go to Q.N. 22	1. Yes<br>2. No	

21	If the answer for Q.N. 20 is “No” then what do you do when the containment is full?	1. Dig/build a new containment<br>2. Continue using and allow it to overflow<br>3. Haven’t emptied yet<br>4. other<br><br>………………..<br>(Please specify)	
22	If the containment is accessible for emptying, how do you empty the containment?	1. Do it myself<br>2. Hire someone with buckets/ shovels to do it (manually)<br>3. Private service provider with mechanical equipment<br>4. Municipality with mechanical equipment	
23	If answer to Q. 22 is “1. Do it myself”, then give reason:	1. High cost of emptying from municipality/ private service providers<br>2. Containment is not easily accessible<br>3. The service provider is not timely available<br>4. No idea as to whom to contact<br>5. Unavailability of service provider<br>6. Other<br>…………..<br>(Please specify)	
24	If the response to Q.N. 22 is “1” or “2” then where do you dispose the sludge emptied from the containment?	1. Agricultural field<br>2. Open area<br>3. Drainage channel	

		4. Others ………………. (Please specify)	
25	How often do you empty the containment?	1. Once in a year<br>2. 2-3 times in a year<br>3. Once in 2-5 years<br>4. Once in 6-10 years<br>5. Once in more than 11 years<br>6. When there's leakage from containment<br>7. Others ………. (Please specify)	
26	Is the containment accessible for emptying even during adverse weather conditions (Eg: heavy rainfall)?	1. Yes<br>2. No	
27	Can you easily open the manhole of the containment at any time of the year (Eg: even during heavy rainfall)?	1. Yes<br>2. No	
If response to Q.N. 22 is "1" then skip Q.N. 28-32			
28	How did you get the contact of the service provider?	1. Neighbor<br>2. Advertisement<br>3. Friends<br>4. Relatives<br>5. Municipality<br>6. Others ………….. (please specify)	
29.	Where is this business located (try to get a precise location if possible)?	………………….	
30	Do you have access to multiple emptying services or just one?	1. Only one<br>2. More than 1	
31	Can you easily access emptying services at any time of the year (e.g. even during the rainy season)?	1. Yes<br>2. No	
32	What do you think about the emptying service charge?	1. Expensive<br>2. Cheap<br>3. Fine	

33	Are there roads or bridges that are frequently damaged or flooded in or around your community?	1. Yes<br>2. No	
	**IV. Questions on community cohesion and engagement**		
34	Have you heard about 'climate change'?<br><br>If response is "2 or 3", go to Q.N.36	1. Yes<br>2. No<br>3. Heard but don't know the meaning	
35	If the response to Q.N. 34 is "Yes", do you think there is a threat from climate change to your latrine or sanitation service?	1. Yes<br>2. No	
36	Which of the following or other climatic events can have an impact on your latrine or sanitation service?	1. Rising groundwater table<br>2. Flood<br>3. Drought<br>4. Heavy rainfall<br>5. Landslide<br>6. Others<br>………………….<br>(Please specify)	
37.	Have you made any changes to your latrine and/or the containment to make it more resilient to flooding or damage from heavy rain or falling debris?<br><br>If response is "No" go to Part II: Site inspection	1. Yes<br>2. No	
37.a	If the response to Q.N. 37 is "Yes", which changes did you make?	1. Sealed the container<br>2. Raised the containment above the surface level<br>3. Raised the toilet above the surface level<br>4. Other<br>…………………<br>(Please specify)	
38.	Did you make any changes/improvements yourself or did you hire someone to do them?	1. Myself<br>2. Hired someone (technician)	
39	How much did you pay for improving the containment or latrine?	……………..	

40	Have you considered that these expenses may increase because of more severe weather in the future?	1. Yes<br>2. No	
**Part II: Site inspection – to be done by the field investigator through visual inspection**			
**V :identify risks in the wider environment (Information from the user collected in questions 1 to be verified through visual inspection)**			
41	Is the latrine located near a surface water body prone to flooding?	1. Yes<br>2. No	
42	Where is the latrine located?	1. Downhill of a steep slope with cultivated land<br>2. Afforested land<br>3. Steep slope with bare soil<br>4. Loose rocks<br>5. Landslide prone<br>6. Others ……………….. (Please specify)	
43	Is there evidence of past landslips e.g. landslip scars, uphill of the household/latrine?	1. Yes<br>2. No	
VI: identify risks at the latrine (Information from the user collected in questions 2-20 to be verified through visual inspection)			
44	Is the latrine raised above the ground?	1. Yes<br>2. No	
45	Is the containment raised above the ground?	1. Yes<br>2. No	
46	Are there cracks in the latrine slab that might allow water to enter the containment unit?	1. Yes<br>2. No	
47.	Are there leaks in the roof of the superstructure that might allow rainwater to enter the containment unit?	1. Yes<br>2. No	
48.	Is the latrine's infrastructure permanent (i.e. well built at least to withstand minor storms and/or heavy rainfalls)?	1. Yes<br>2. No	
49.	Does the containment unit have an outlet?<br>If the response is "No" go to Q.N.51	1. Yes<br>2. No	
50.	If the answer is **yes** for Q.N. 50, where is the outlet connected to?	1. Open area<br>2. Drainage or channel<br>3. Agricultural land<br>4. Other<br>…………………..<br>Please specify	

51.	Is the manhole of the containment accessible for regular pumping or emptying?	1. Yes<br>2. No	

Table S1. Households and their sanitation characteristics by countries

Variable	Count (%) / Mean value			
	Total Sample	Ethiopia	Uganda	Nepal
**Gender of respondent**				
Male	405 (55.2%)	116 (54%)	108 (35.4%)	181 (84.6%)
Female	329 (44.8%)	99 (46%)	197 (64.6%)	33 (15.4%)
**Type of employment of respondent**				
Not formally employed (e.g. farmer, student, unemployed, homemaker, etc.)	264 (36.4%)	121 (56.3%)	116 (39.1%)	27 (12.6%)
Formally employed (e.g. government employee, private employment, etc.)	462 (63.6%)	94 (43.7%)	181 (60.9%)	187 (87.4%)
**Education of respondent**				
Completed secondary/high school level or higher	360 (50.1%)	81 (37.7%)	220 (75.9%)	59 (27.6%)
Primary level education or less	359 (49.9%)	134 (62.3%)	70 (24.1%)	155 (72.4%)
**Respondent familiar with the term "climate change"**				
Yes	428 (58.2%)	105 (48.8%)	250 (81.4%)	73 (34.1%)
No	308 (41.8%)	110 (51.2%)	57 (18.6%)	141 (65.9%)
**Fully sealed containment unit**				
Yes	75 (10.3%)	0 (0%)	63 (20.9%)	12 (5.61%)
No	655 (89.7%)	215 (100%)	238 (79.1%)	202 (94.39%)
**Owner has paid to make latrine more resilient**				
Yes	169 (23.6%)	52 (24.2%)	98 (34%)	19 (8.88%)
No	548 (76.4%)	163 (75.8%)	190 (66%)	195 (91.12%)
**Proximity of latrine to flood-prone water body**				
Yes	228 (31.1%)	33 (15.3%)	65 (21.4%)	130 (60.7%)
No	505 (68.9%)	182 (84.7%)	239 (78.6%)	84 (39.3%)
**Toilet is raised above the ground**				
Yes	415 (56.7%)	6 (2.79%)	257 (84.8%)	152 (71%)
No	317 (43.3%)	209 (97.21%)	46 (15.2%)	62 (29%)

**Presence of cracks in latrine slab**				
Yes	101 (13.8%)	67 (31.2%)	18 (5.96%)	16 (7.48%)
No	630 (86.2%)	148 (68.8%)	284 (94.04%)	198 (92.52%)
**Presence of leaks in superstructure roof**				
Yes	204 (27.8%)	79 (36.7%)	64 (21.1%)	61 (28.5%)
No	529 (72.2%)	136 (63.3%)	240 (78.9%)	153 (71.5%)
**Presence of permanent latrine superstructure**				
Yes	563 (77.4%)	106 (49.3%)	285 (95.6%)	172 (80.4%)
No	164 (22.6%)	109 (50.7%)	13 (4.4%)	42 (19.6%)
**Location of household**				
Peri-urban or urban	620 (84.0%)	161 (74.9%)	309 (100%)	150 (70.1%)
Rural	118 (16.0%)	54 (25.1%)	0 (0%)	64 (29.9%)
**Number of households in Ethiopia**	215 (29.1%)	215 (100%)	0 (0%)	0 (0%)
**Number of households in Uganda**	309 (41.9%)	0 (0%)	309 (100%)	0 (0%)
**Number of households in Nepal**	214 (29.0%)	0 (0%)	0 (0%)	214 (100%)
**Number of households in Terai region of Nepal**	120 (16.3%)	0 (0%)	0 (0%)	120 (56.1%)
**Mean age of respondent**	41	45.30	33.56	45.99
**Mean number of users per toilet**	12	8.64	18.87	5.78
**Mean family size**	5	5.48	N/A	4.96
